# Supplementary material for: A paradoxical misperception of relative motion
Source: Proc Natl Acad Sci U S A. 2024 Nov 21;121(48):e2410755121. doi: 10.1073/pnas.2410755121 (PMC11621632; doi:10.1073/pnas.2410755121)
Supplement: Supplementary file 1 — Appendix 01 (PDF) [file pnas.2410755121.sapp.pdf]

PNAS

Supporting Information for

A paradoxical misperception of relative motion

Josephine C. D'Angelo, Pavan Tiruveedhula, Raymond J. Weber, David W. Arathorn, and Austin Roorda

Austin Roorda.
E-mail: aroorda@berkeley.edu

This PDF file includes:

Supporting text
Figs. S1 to S6

Supporting Information Text

1. Description of Retina-contingent Stimulus Delivery

This section explains the mechanics of how retina-contingent stimuli are projected onto the retina within the raster-scan of the AOSLO. Note that the raster-scan is always world-fixed so motion of a stimulus in the raster is equivalent to motion in the world.

Definitions.

- Gain: the amplitude of the motion of the projected stimulus relative to the eye motion.
- Angle: the angle of the motion of the projected stimulus relative to the eye motion.

Examples.

- Gain 1 and Angle 0 defines a stabilized stimulus on the retina. The stimulus moves in the raster by an amount that is identical to the eye motion.
- Gain 1 and Angle 180 defines a stimulus that moves in the raster by an amount that is identical to the eye motion, but in the opposite direction. It moves with double the retinal slip across the retina. For simplicity, this is stated in the main manuscript as Gain = −1.
- Gain 1 and Angle 90 defines a stimulus that moves in the raster by an amount that is identical to the eye motion, but rotated by 90°.

For a raster-scanned stimulus whose location is retina-contingent, the process is done in the following way. There is an FPGA board installed in the system computer whose inputs are image data from the AOSLO and whose outputs drive the acousto-optic laser modulators that project the stimulus onto the retina. There is a memory buffer in the FPGA that stores the acousto-optic modulator playout signal that encodes the stimulus. The memory buffer is stored as a 2-D array representing a complete image frame. (NOTE: On the system computer, the stimulus and its location are represented in linear spatial coordinates but there is a transformation between this and the playout memory buffer on the FPGA that accounts for the non-linear sinusoidal scanning of the resonant (fast) scanner. The transformation is done through a lookup table that is generated with a system calibration prior to the experiment.) A reference frame is established prior to initiation and delivery of a retina-contingent display. A stimulus location is identified within the reference frame. This location is set by either a mouse/cursor click or programmed to have a predetermined coordinate (e.g. pixel location 256, 256). The stimulus (e.g. letter 'E') is initially placed in the playout buffer at this raster location. The AOSLO image data is retrieved from the FPGA board in the form of image strips that comprise the full width of the image and a predetermined height. Typically, in 30 Hz mode the strip height is 16 pixels and there are 32 strips per 512 x 512 frame. In 60 Hz mode, the strip height is 16 pixels and there are 16 strips per frame. The location of each strip is found relative to the reference frame and the $\triangle$X and $\triangle$Y offsets required for best registration indicate the new position of the retina relative to the reference frame. The $\triangle$X, $\triangle$Y, Gain, & Angle are used to compute the new position of the stimulus. For example, for [Gain = 1, Angle = 90] if the eye has been found to be shifted to the right by 5 pixels (~1 arcminute) relative to the reference frame, then the new position of the stimulus in the playout buffer should be up in the raster by 5 pixels. If in the next frame the eye has been found to be shifted to the right by 10 pixels relative to the reference frame, then the new position of the stimulus in the playout buffer is moved up in the raster by 10 pixels. This recomputing of the stimulus position in the playout buffer is done for each strip but the stimulus location in the memory buffer is only updated once per frame when it is determined that the target location will soon be swept by the scanning raster. This once-per-frame update is done to overcome bandwidth limitations (minimize bottlenecks) of data transfer between the CPU and the FPGA. This is OK, since the stimulus in the buffer will not be played out until the raster scans over the location, which also occurs just once per frame. Due to the high-speed processing, the updates of stimulus position are generally made just 2-3 milliseconds prior to the raster passing over the target. It is this low latency that gives the AOSLO its impressive targeting accuracy.

2. Control Experiments

A. Method of adjustment with two parameters to adjust. To explore the accuracy with which a subject matches two motion trajectories, we performed a method of adjustment experiment with two adjustable parameters: the α and the diffusion constant. The experiment ran as follows, the subject fixated on a fixation target and attended to two stimuli presented over intervals in time. Both stimuli moved on pre-programmed random trajectories, independent to eye motion. The stimulus in the first interval moved with a selected α and diffusion constant. The subject's task was to adjust the α and diffusion constant of the stimulus in the second interval until its motion looked perceptually equivalent to that of the stimulus in the first interval. The subject made their adjustments on a gamepad. Increasing the α parameter greater than one led to more persistent trajectories while decreasing less than one led to more antipersistent trajectories. The α parameter set to one displayed pure random trajectories. Increasing the diffusion constant led to longer path lengths and decreasing the diffusion constant led to shorter path lengths. We tested seven α and diffusion constant combinations: 0.7 and 3.2 arcmin^2/s, 0.9 and 1.8 arcmin^2/s, 1.0 and 4.8

arcmin^2/s, 1.0 and 14.7 arcmin^2/s, 1.2 and 24.5 arcmin^2/s, 1.5 and 58.1 arcmin^2/s, and 1.7 and 84.3 arcmin^2/s. We repeated each match two times.

Fig. S3 plots the random walks' diffusion constants as a function of the α's. The seven black circles represent the tested α's and diffusion constants and the small colored circles represent the subject's perceptual matches. For matches that deviated from the tested α and diffusion constant, the data points are connected to the respective black circle with a line of the same color. We found that sometimes the subject would correctly match the α and diffusion constant, while for other matches the values were both much higher or both much lower. For example, under background present conditions with tested α and diffusion constant equal to 1.7 and 84.3 arcmin^2/s, respectively, the subject reached a perceptual match at these values as well as at 1.5 and ~70 arcmin^2/s (Fig. S3, red). What could contribute to this mismatch, is that random walks with high α and diffusion constant values can be generated from the same average step length as random walks with low α and diffusion constant values, examples are shown in Fig. 1c. These results motivated us to constrain the random walk stimulus' α in the main experiments to be equal to one.

Methods: How we generated random walks with varying α's and diffusion constants. Prior to the experiment, we pre-programmed 100s of 750-ms and 1500-ms duration random walks with varying α's and varying diffusion constants for the subject to use in the matching task. The α's ranged from 0.7 to 1.9 in increments of 0.1. These paths were generated by computing randomized step lengths in x and y drawn from a normal distribution with a range of standard deviations from 0.05 to 1.6 arcminutes per step (or AOSLO frame), in increments of 0.05 arcminutes. For the sake of brevity, we will refer to the standard deviation of the randomized step lengths as simply *step lengths.* We converted the step lengths to polar coordinates to obtain the 2-dimensional step. Next, we selected the angle. All angles were equally probable for the first step. For all subsequent steps, when generating paths with α greater than one, each angle was drawn from a normal distribution with a mean equal to the angle of the previous step and with a standard deviation determined by the α. (When generating paths with α less than one, for all subsequent steps, each angle was drawn from a normal distribution with a mean equal to the angle of the previous step subtracted from 180° and with a standard deviation determined by the α.) The more the α deviated from one, the smaller the standard deviation. After generating all 2D steps and angles for the particular duration, we converted back to Cartesian coordinates by taking the cosine and sine of the angles and multiplying by the 2D steps to determine the x and y positions, respectively. The positions were successively added to generate the trajectory.

This process effectively randomized the length of each step. The direction was also randomized; however, as α deviated greater than one, the trajectory more likely moved in the same direction as the previous step; and, as α deviated less than one, the trajectory more likely moved in the opposite direction of the previous step. Owing to the random nature of generating paths in this method, the same randomized step length could generate a path with a range of possible diffusion constants and α's. We computed the average diffusion constant for each step length and selected α, by inputting the step length as the $\triangle$X and $\triangle$Y in the mean square displacement formula in equation 2. After solving for the mean square displacement, we input this value as well as the selected α into equation 1 to solve for the diffusion constant. For example, a step length of 1.6 arcminutes and α equal to 1.2 corresponded to a diffusion constant of 174.2 arcmin^2/s. Therefore, for each of the 32 step lengths, we generated 100 paths and from these, we selected the 10 paths with diffusion constants closest to the respective average diffusion constant and α's closest to the selected α.

We used this method to also generate 50 trajectories for each selected α and diffusion constant tested. For each presentation, one of the 50 paths were selected randomly to present in the first interval.

B. Method of adjustment with a stimulus that moves orthogonal to eye motion with Gain +1.5. We performed a control experiment for one subject with the same method of adjustment protocol, except that we replaced the Gain 0 stimulus with a stimulus that moved orthogonal to eye motion with a Gain of +1.5. For brevity, we will refer to this stimulus as the *90° stimulus*. We expect that subjects will perceive motion as long as the stimulus moves in any direction other than in a direction consistent with retinal slip. If this is true, then the 90° stimulus would be perceived as moving similar to the Gain +1.5 stimulus.

World motion of the retina contingent stimuli. Undergoing the same retinal motion, the Gain −1.5, Gain +1.5, and 90° stimuli move equally in the world. Said another way, the D_{WM} of the Gain −1.5, Gain +1.5, and 90° stimuli are equal. However, note that these stimuli move in different directions: the Gain −1.5 and Gain +1.5 move opposite to each other, and the 90° stimulus moves orthogonal to both.

Retinal motion of the retina contingent stimuli. Undergoing the same retinal motion, the Gain −1.5, Gain +1.5, and 90° stimuli move differently on the retina. A Gain −1.5 stimulus moves across the retina with 2.5x more motion than a natural, world-fixed stimulus. A Gain +1.5 stimulus has half the retinal motion of a world-fixed stimulus. A 90° stimulus moves with $(\sqrt{(1.5^2 + 1^2)})$x more retinal motion than a world-fixed stimulus. For example, if the retina moved 1' right, the stimulus would move 1.5' up in the world. The retinal motion would be the Euclidean distance. The Gain −1.5 stimulus would have the highest retinal motion (D_{RM}), followed by a 90° stimulus, followed by a world-fixed stimulus, followed by a Gain +1.5 stimulus.

Results. Fig. S4 plots the diffusion constants of the retina contingent stimulus' perceived motion (D_{PM}) as a function of the world motion (D_{WM}). The red arrows show the extent to which the eye motion, and consequent retina-contingent stimulus' world motion (α_{WM}), deviated from Brownian. Arrows pointing right indicate persistence ($\alpha_{WM} > 1$), arrows pointing left indicate antipersistence ($\alpha_{WM} < 1$), and no arrow means that the motion was Brownian (α_{WM} = 1 +/− 0.02). Under

background-present conditions (Fig. S4, left), a paired t-test revealed no significant difference in the perceived motion between the Gain +1.5 stimulus and 90° stimulus ($p = 0.18$). Both perceived motions were significantly greater than that of the Gain −1.5 stimulus ($p = 0.0031$ Gain +1.5; $p = 0.00030$ 90° stimulus). Yet, the trends were different under background-absent conditions (Fig. S4, right). The Gain −1.5 stimulus now appeared to be moving the most, followed by the 90° stimulus, followed by the Gain +1.5 stimulus. The perceived motion of the Gain −1.5 stimulus was significantly greater than that of the 90° stimulus ($p = 0.02$, paired t-test). The perceived motion of the 90° stimulus was significantly greater than that of the Gain +1.5 stimulus ($p = 0.01$, paired t-test).

C. Method of adjustment with 750-ms duration intervals under background-present conditions. In the main study, the stimuli were presented for 1500-ms under background-present conditions and for 750-ms under background-absent conditions. We performed a control experiment for two subjects with the same method of adjustment protocol, except that we presented the stimuli for a 750-ms duration under background-present conditions. This experiment verified that the different durations did not contribute significantly to the results. For each subject, a paired t-test comparing the diffusion constants for perceived motion (D_{PM}) revealed that, similar to the 1500-ms duration results, stimuli moving in advance of eye motion (Fig. S5, Gain +1.5) were seen as moving significantly more than stimuli moving in a direction consistent with retinal slip (Fig. S5, Gain −1.5) (10003L: $p = 0.0057$ and 20114R: $p = 0.042$) .

D. Method of adjustment with the fixation cross remaining on during the random walk interval under background-absent conditions. In the original experiments tested under background-absent conditions, the fixation target was turned off during both the retina-contingent interval and the random walk interval. Because there was no frame of reference during the random walk interval, we could not truly assess the amount of motion perceived. Specifically, we could not conclude that the subject did not perceive motion when they matched a Gain 0 stimulus to a non-moving stimulus in the second interval. We performed a control experiment for one subject with the same method of adjustment protocol, except that the fixation target remained on to serve as a frame of reference during the second interval where they made the match (Fig. S6b).

We confirmed that the subject perceived almost no motion when they viewed a Gain 0 stimulus under background-absent conditions (Fig. S6a). A paired t-test comparing the Gain 0 diffusion constants for perceived motion (D_{PM}) revealed no significant differences whether the fixation target was on (Fig. S6a) or off (Fig. S1, 10003L background-absent) during the random walk interval ($p = 0.78$).

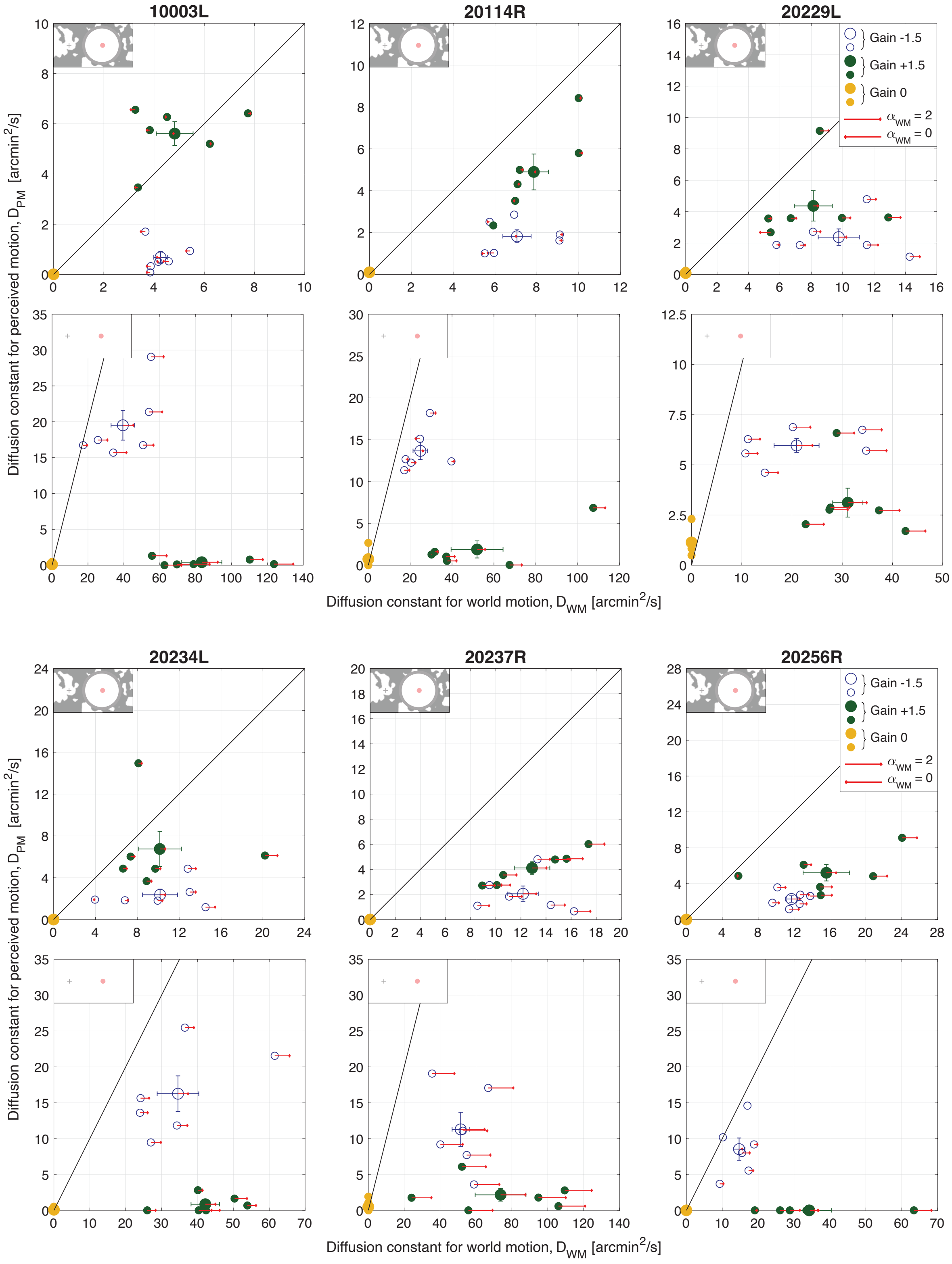

Fig. S1. Individual results from six subjects tested under background-present and background-absent conditions. The conditions are indicated by labels on the top left corner of each graph. The small circles are the six perceptual matches for Gain -1.5 stimuli (blue open symbols), Gain $+1.5$ stimuli (green filled symbols), and Gain 0 stimuli (yellow filled symbols). The large circles are the averages of the matches with standard error of the mean bars. The red arrows show the extent to which the eye motion, and consequent retina-contingent stimulus' world motion (α_{WM}), deviated from Brownian. Arrows pointing right indicate persistence ($\alpha_{WM} > 1$), arrows pointing left indicate antipersistence ($\alpha_{WM} < 1$), and no arrow means that the motion was Brownian ($\alpha_{WM} = 1$ +/− 0.02). Longer arrows correspond to higher deviations from Brownian motion. The arrow length in the legend indicates pure persistence ($\alpha_{WM} = 2$, straight line trajectory at constant velocity) if pointing right or pure antipersistence ($\alpha_{WM} = 0$, oscillatory motion) if pointing left.

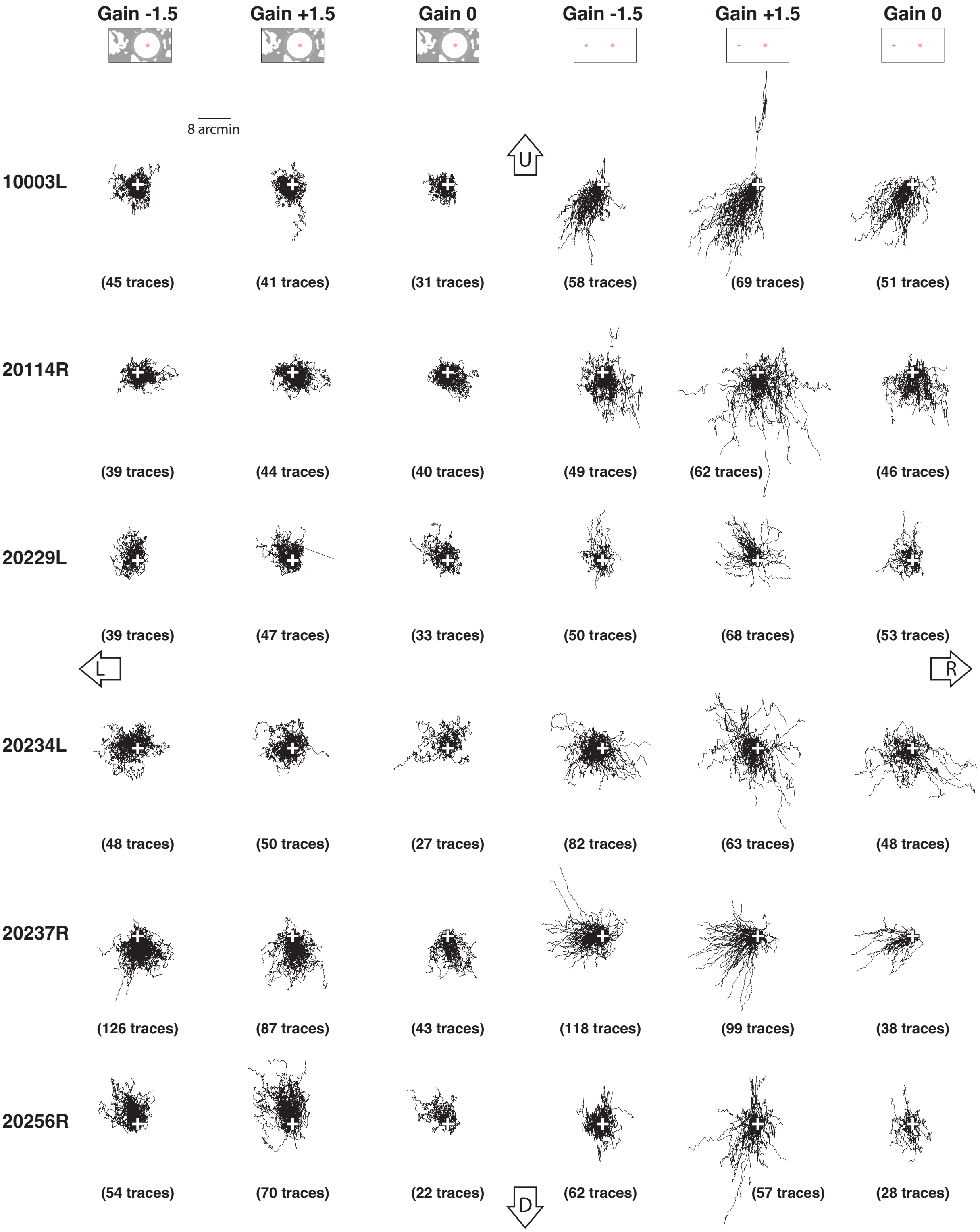

Fig. S2. Gaze traces for six subjects for the three Gain conditions (-1.5, $+1.5$, and 0) under background-present (1500-ms duration) and background-absent (750-ms duration) conditions. The Gains and background conditions are indicated by labels at the top of the figure. The white cross indicates the starting position for each trace. The gaze directions are labeled: left (L), right (R), up (U), and down (D).

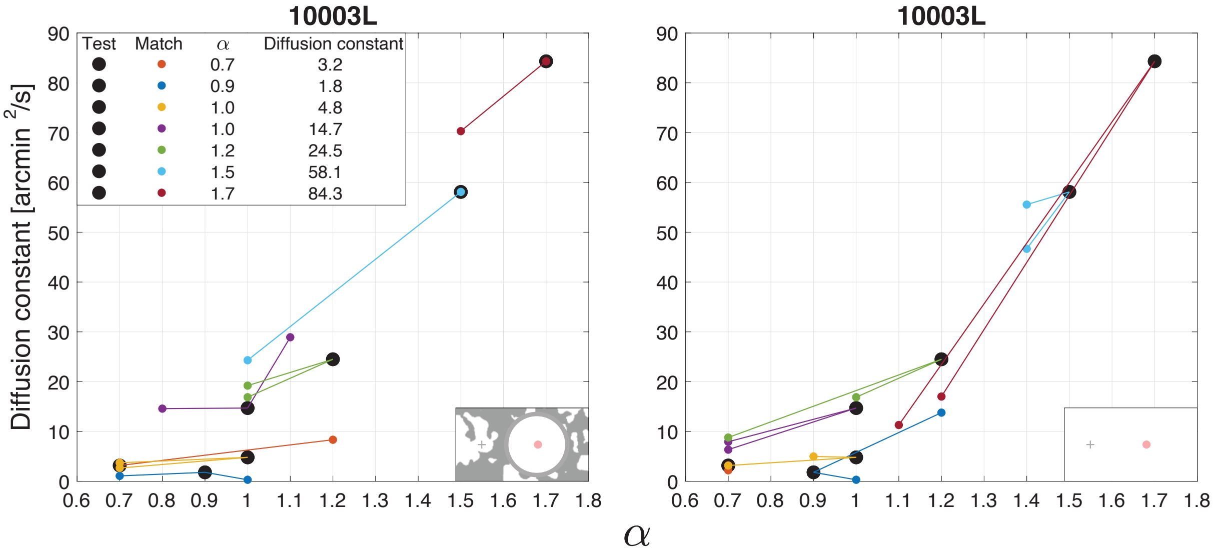

Fig. S3. Matches from one subject tested under background-present and background-absent conditions. The conditions are indicated by labels on the bottom right corner of each graph. The large black circles are the tested α and diffusion constant values: 0.7 and 3.2 arcmin^2/s, 0.9 and 1.8 arcmin^2/s, 1.0 and 4.8 arcmin^2/s, 1.0 and 14.7 arcmin^2/s, 1.2 and 24.5 arcmin^2/s, 1.5 and 58.1 arcmin^2/s, and 1.7 and 84.3 arcmin^2/s. The small circles are the two perceptual matches for each tested α and diffusion constant value.

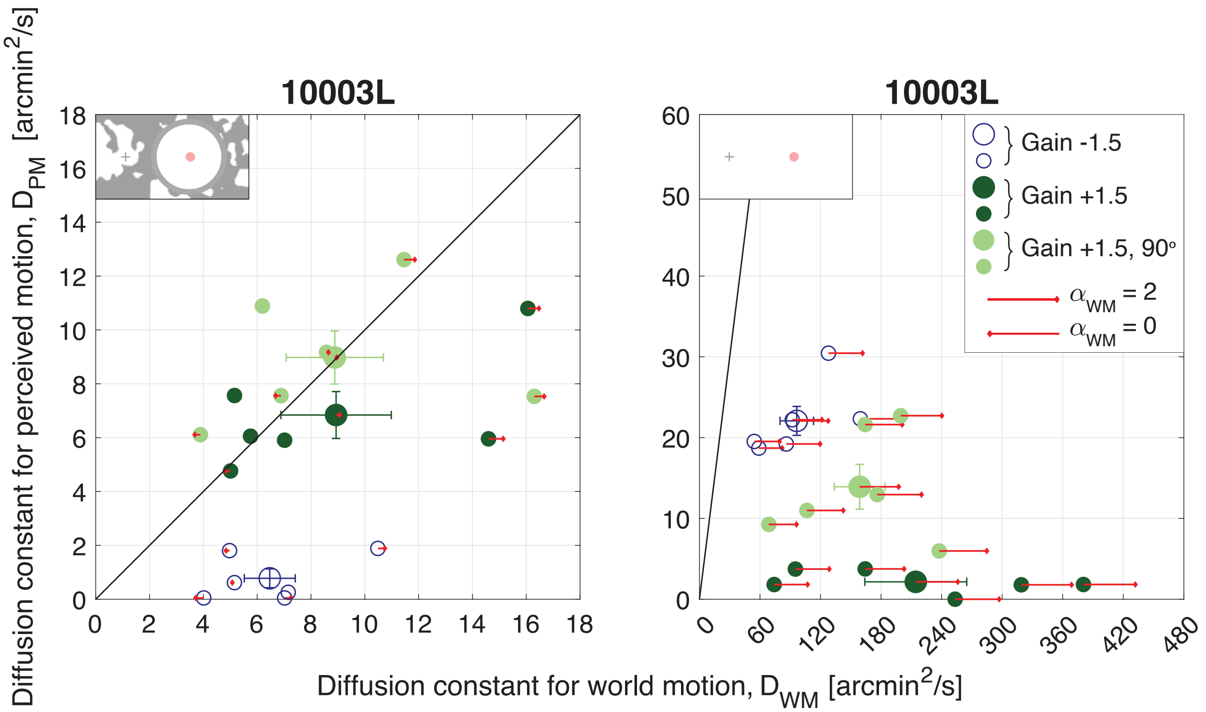

Fig. S4. Individual results from one subject tested under background-present and background-absent conditions. The conditions are indicated by labels on the top left corner of each graph. The small circles are the six perceptual matches for Gain -1.5 stimuli (blue open symbols), Gain $+1.5$ stimuli (dark green filled symbols), and Gain $+1.5$, 90° stimuli (light green filled symbols). The large circles are the averages of the matches with standard error of the mean bars. The red arrows show the extent to which the eye motion, and consequent retina-contingent stimulus' world motion (α_{WM}), deviated from Brownian. Arrows pointing right indicate persistence ($\alpha_{WM} > 1$), arrows pointing left indicate antipersistence ($\alpha_{WM} < 1$), and no arrow means that the motion was Brownian ($\alpha_{WM} = 1 +/- 0.02$). Longer arrows correspond to higher deviations from Brownian motion. The arrow length in the legend indicates pure persistence ($\alpha_{WM} = 2$, straight line trajectory at constant velocity) if pointing right or pure antipersistence ($\alpha_{WM} = 0$, oscillatory motion) if pointing left.

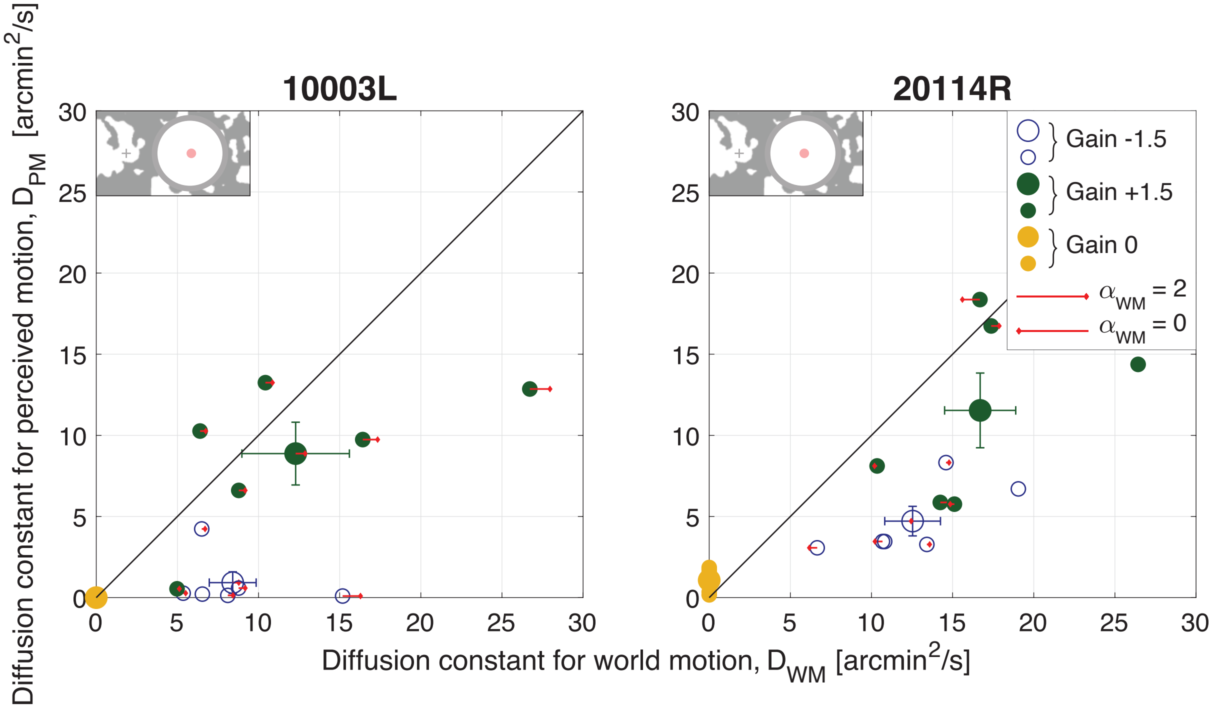

Fig. S5. Individual results from two subjects tested under background-present conditions with 750-ms stimulus duration intervals. The condition is indicated by labels on the top left corner of each graph. The small circles are the six perceptual matches for Gain -1.5 stimuli (blue open symbols), Gain $+1.5$ stimuli (green filled symbols), and Gain 0 stimuli (yellow filled symbols). The large circles are the averages of the matches with standard error of the mean bars. The red arrows show the extent to which the eye motion, and consequent retina-contingent stimulus' world motion (α_{WM}), deviated from Brownian. Arrows pointing right indicate persistence ($\alpha_{WM} > 1$), arrows pointing left indicate antipersistence ($\alpha_{WM} < 1$), and no arrow means that the motion was Brownian ($\alpha_{WM} = 1 +/- 0.02$). Longer arrows correspond to higher deviations from Brownian motion. The arrow length in the legend indicates pure persistence ($\alpha_{WM} = 2$, straight line trajectory at constant velocity) if pointing right or pure antipersistence ($\alpha_{WM} = 0$, oscillatory motion) if pointing left.

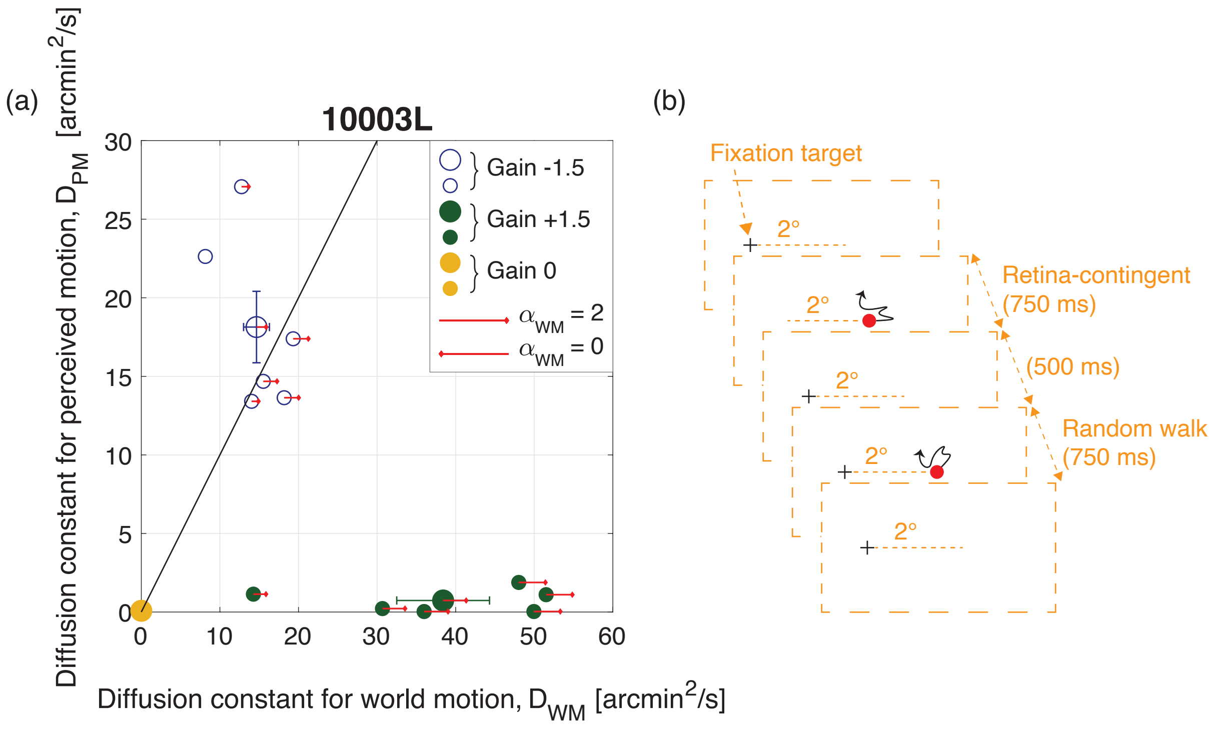

Fig. S6. Control experiment tested under background-absent conditions. (a) Results from one subject. The small circles are the six perceptual matches for Gain -1.5 stimuli (blue open symbols), Gain $+1.5$ stimuli (green filled symbols), and Gain 0 stimuli (yellow filled symbols). The large circles are the averages of the matches with standard error of the mean bars. The red arrows show the extent to which the eye motion, and consequent retina-contingent stimulus' world motion (α_{WM}), deviated from Brownian. Arrows pointing right indicate persistence ($\alpha_{WM} > 1$), arrows pointing left indicate antipersistence ($\alpha_{WM} < 1$), and no arrow means that the motion was Brownian ($\alpha_{WM} = 1$ $+/-$ 0.02). Longer arrows correspond to higher deviations from Brownian motion. The arrow length in the legend indicates pure persistence ($\alpha_{WM} = 2$, straight line trajectory at constant velocity) if pointing right or pure antipersistence ($\alpha_{WM} = 0$, oscillatory motion) if pointing left. (b) Experimental sequence. The fixation target remained on during the random walk interval.
